# Treatment of Fractures by Massage

**Published:** 1888-02

**Authors:** F. A. Harrington

**Affiliations:** 342 Fourteenth street; Assistant to the Chair of Materia Medica, Niagara University


### Translations.

### *TREATMENT OF FRACTURES BY MASSAGE.*

BY DR. BERNE.

Translated from *Journal de Médicine de Paris*, by F. A. HARRINGTON, M. D., Assistant to the Chair of Materia Medica, Niagara University.

Various sessions of the Surgical Society have been devoted, in part, to the question of treatment of fractures by massage. Having an experience of four years in this method of practicing surgery, I think it is a duty to relate my personal opinion on the subject. In relation to fractures of the fibula, the priority of research on that special subject is mine. I performed, in the service of Professor Duplay, then in the hospital at Lariboisière, a series of experiments in this particular mode of treatment. The preceding year, I had treated one of my patients, examined by Doctors Love and Coupard, employing, from the first day, massage for the treatment of a fracture of the fibula, situated two centimeters above the external malleolus. The patient was able to walk perfectly on the seventieth day.

It is important to determine the indications and the conditions for this course of treatment.

First, what fractures are with propriety treated by massage? They are the fractures near the ends of limbs, which have two bones, the leg and forearm. Nor must we forget to describe another class of fractures: that of the patella and olecranon. As regards the radius, all the fractures of its lower extremity cannot be treated by massage at the beginning, but all should be on the fifteenth day. In case of the fibula, massage can be practiced early, when there is opposition of the fragments.

Secondly, is it necessary to immobilize the fractured limb, or to leave it free?

Lucas-Championnière uses no apparatus; but my own personal experience teaches that, although you can massage certain fractures of the radius without fixation, you should combine fixation and massage, by using some appliance in the treatment of fractures of the fibula. At the time of my investigations at Lariboisière, in 1885, the fractures were put up in plaster splints, which were applied simply posterior, and fastened by three or four circular bands, furnished with buckles, and easily removed at each visit. Massage was performed every second day, for ten minutes, and the cure was complete in from fifteen to seventeen days, under the treatment. Unfortunately, my observations remained unpublished, I regret to say; not that I might claim priority, but as proof of my theory. Massage should not be practiced, to begin with, only in case of fractures without displacement; and it is a great advantage to have the limb in an apparatus that can be removed.

Massage is performed, not to render the formation of the callus more active, but to favor the absorption of extravasated blood, and to prevent adhesion of the tendons. It is necessary to massage the tibio-tarsal articulation in fractures of the fibula, and, in fractures of the radius, the small carpal joints. Shedė, of Hamburg, and Neutzel, of Trieste, early employ massage and immobilization.

Massage must not be employed, to begin with, whenever there is any displacement; otherwise, it should be done at once. In a fracture of the radius, you will not make active motion, but merely passive, during the first week; afterwards, motion of any sort whatever can be made.

In fractures of the patella, massage should be practiced immediately.

To resume, in the treatment of fractures by massage, your aim is, at first, to favor the absorption of extravasated blood, but you have nothing to do with the fractured bone, for the callus forms of itself. It is necessary to immobilize the joints adjacent to the fracture.

The kneading of the muscles of the calf and the forearm favors the lymphatic and the venous circulation. In this way, the normal temperature is preserved, whereas, if the limbs are immobilized for a long time by an apparatus, there is a marked lowering of temperature.

The results of my experiments have been published.

Massage should be performed according to certain rules, depending on age and on the requirements of each case. It should be done by a person who is skilled in its performance; it needs to be carefully practiced, and then only a short time, otherwise it is useless and injurious. It is a profound mistake to think it is a gain to massage a limb for more than ten or twenty minutes.

To summarize, massage is applicable to fractures of the extremities of bones near the joints, taking into consideration the age of the patient, the diathesis, rheumatic, etc., and the age of the fracture.

It should be performed for fifteen minutes at the most, and once a day, or every alternate day. If the attending physician is unable to perform massage himself, he should preferentially employ some older method of treatment rather than place his patient in the hands of an assistant who is unskillful or rough.

342 Fourteenth street.

